# Multistable Synaptic Plasticity Induces Memory Effects and Cohabitation of Chimera and Bump States in Leaky Integrate-and-Fire Networks

**DOI:** 10.3390/e27030257

**Published:** 2025-02-28

**Authors:** Astero Provata, Yannis Almirantis, Wentian Li

**Affiliations:** 1 Institute of Nanoscience and Nanotechnology, National Center for Scientific Research “Demokritos”, 15341 Athens, Greece; 2 Institute of Biosciences and Applications, National Center for Scientific Research “Demokritos”, 15341 Athens, Greece; yalmir@bio.demokritos.gr; 3 Department of Applied Mathematics and Statistics, Stony Brook University, Stony Brook, NY 11794, USA; wtli2012@gmail.com; 4 The Robert S. Boas Center for Genomics and Human Genetics, The Feinstein Institutes for Medical Research, Northwell Health, Manhasset, NY 11030, USA

**Keywords:** synchronization, bistable plasticity, synaptic plasticity, weighted network, adaptive network, chimera-like states, bump-like states, memory effects, confinement, entropy deviation

## Abstract

Chimera states and bump states are collective synchronization phenomena observed independently (in different parameter regions) in networks of coupled nonlinear oscillators. And while chimera states are characterized by coexistence of coherent and incoherent domains, bump states consist of alternating active and inactive domains. The idea of multistable plasticity in the network connections originates from brain dynamics where the strength of the synapses (axons) connecting the network nodes (neurons) may change dynamically in time; when reaching the steady state the network connections may be found in one of many possible values depending on various factors, such as local connectivity, influence of neighboring cells etc. The sign of the link weights is also a significant factor in the network dynamics: positive weights are characterized as excitatory connections and negative ones as inhibitory. In the present study we consider the simplest case of bistable plasticity, where the link dynamics has only two fixed points. During the system/network integration, the link weights change and as a consequence the network organizes in excitatory or inhibitory domains characterized by different synaptic strengths. We specifically explore the influence of bistable plasticity on collective synchronization states and we numerically demonstrate that the dynamics of the linking may, under special conditions, give rise to co-existence of bump-like and chimera-like states simultaneously in the network. In the case of bump and chimera co-existence, confinement effects appear: the different domains stay localized and do not travel around the network. Memory effects are also reported in the sense that the final spatial arrangement of the coupling strengths reflects some of the local properties of the initial link distribution. For the quantification of the system’s spatial and temporal features, the global and local entropy functions are employed as measures of the network organization, while the average firing rates account for the network evolution and dynamics. In particular, the spatial minima of the local entropy designate the transition points between domains of different synaptic weights in the hybrid states, while the number of minima corresponds to the number of different domains. In addition, the entropy deviations signify the presence of chimera-like or bump-like states in the network.

## 1. Introduction

During the past two decades considerable efforts have been devoted to the understanding of complex synchronization phenomena observed in networks of coupled nonlinear oscillators. Such phenomena include traveling waves, spiral (2D) and scroll (3D) waves, chimera states and bump states to name just a few [1,2,3,4,5]. In particular, the phenomena of hybrid synchronization (chimera states or bump states) were unexpected since they were first shown to occur in networks consisting of identical elements with identical linking between nodes and were, therefore, associated with spatial symmetry breaking in the network [6,7]. While in the earlier works the connectivity in the system was considered to be constant/uniform or random or statistically homogeneous, in recent years the research interest has shifted toward temporal and adaptive networks whose link weights depend on time and they may gradually develop heterogeneous connectivity properties. Inspired by the recent advances in the domain of temporal neural networks we here investigate the influence of link plasticity on the emergence and the form of hybrid synchronization patterns.

From the point of view of applications, the studies of coupled nonlinear oscillators are motivated by brain dynamics, where neural cells operate as potential integrators and they connect with other neural cells via their axons forming large complex networks [8,9,10,11] (Note that there are also neural cells that operate as resonators and only respond to inputs with well defined frequencies (resonate at specific frequencies) [12]. These are outside the scope of this work). The intricate structure of these networks allows the propagation of information in the brain for the control and regulation of a wide variety of body and cognitive functions such as sensory processes, motor actions, thinking, problem-solving, reasoning, learning, memory, emotions, consciousness, homeostasis and many others [13,14,15,16].

Recent studies of the network structure of the human brain have shown that the connectivity between neural cells is not static but dynamical and changes with time due to various factors, such as brain development, aging, diseases, need to adapt to a constantly changing potential environment or modifications based on recent patterns of activity [17]. The connectivity adjustments take place in various time scales. “Short-term plasticity” refers to changes taking place in small time scales (milliseconds to seconds) and is roughly captured in functional connectivity. Functional neuroimaging methods, such as fMRI, MEG or EEG, measure brain activity (indirectly via the blood-oxygen-level-dependent (BOLD) signal in fMRI, or more directly electrical/magnetic fields in EEG, MEG and other electrophysiological methods). From these measures of activity, functional connectivity can be estimated as correlation or synchronization of time series. And although functional connectivity is used as representation of short-term plasticity, the exact relationship between the two is not straightforward. On the other hand, “long-term plasticity” (which includes persistent processes such “long-term potentiation” and “long-term depression”) takes place in longer time scales, typically from minutes to hours but can last much longer. Experimentally, this long-term plasticity is mainly estimated using 3D DTI-MRI techniques: from the 3D images fractional anisotropy can be measured which is then used to estimate the neural connectivity. In even longer time scales (days to weeks or longer) structural plasticity takes place. This regards physical changes in the neural structure, such as formation, elimination, or remodeling of synapses and dendrites. Structural plasticity often builds on long-term synaptic plasticity and is also estimated via 3D imaging techniques. For reviews and studies on long and short term plasticities see [18,19,20,21,22].

With respect to time-dependent plasticity, the adjustment of the weights to the local potential environment or to external stimuli is regarded as a learning processes, meaning that the parameters (connectivity weights) in the network get modified to optimize the organism’s ability to survive, to copy with new challenges, to develop and to respond to life’s demands in general. (As will be discussed, in the present work plasticity will be considered with respect to the local weight (synaptic) environment and not to the local potential environment used often in the literature under the name of adaptivity.) Besides their relevance in life sciences, temporal and adaptive neural networks are now intensively studied in connection with the recent advances in machine learning and artificial intelligence [23,24]. Motivated by the plasticity of the connectivity weights in brain dynamics, we study here the influence of time-dependent coupling in the formation of hybrid synchronization states, such as chimera states and bump states, using as exemplary dynamics the leaky Integrate-and-Fire (LIF) model. To facilitate comparisons between LIF networks with and without plasticity, we have included at the end of this work two related appendices that provide descriptive definitions and examples of chimera and bump states in LIF networks without plasticity.

When studying time-dependent connectivity, we keep in mind that heterogeneity could be established in the coupling weights of the network, even if we start from homogeneous or uniformly randomly distributed initial weights. As the original definition of chimera states is based on identical dynamics and identical linking of all nodes [1,6,7], the patterns resulting due to time-dependent connectivity, if composed by co-existing of coherent and incoherent domains in networks with heterogeneous weights, will be called “chimera-like” states. Likewise,“bump-like” states will be states composed by coexisting active and subthreshold (silent) domains observed in temporal networks where the linking between the nodes may not be homogeneous.

Previews studies of complex synchronization phenomena in LIF networks have revealed the presence of chimera states in a 1D ring, 2D toroidal and 3D hypertoroidal geometries under nonlocal connectivity and for relatively large values of the coupling ranges. Studies on 1D ring geometries demonstrate that chimera and multichimera states (chimera states composed by multiple coherent and incoherent domains) are possible depending on parameters such as coupling range and coupling strength and on the connectivity schemes (including hierarchical, fractal connectivities) [25,26,27,28,29,30]. In 2D toroidal geometries, striking chimera patterns were revealed in the form of coherent or incoherent spots, stripes, grids of spots, spiral waves and other composite patterns [3,31]. In 3D hypertorus geometries the chimera forms were generalized to coherent and incoherent spheres, cylinders, planes and composite patterns [32]. Concerning the presence of bump states in LIF networks, traveling and spatially confined bumps have been reported in 1D and 2D geometries. The multiplicity of bumps, their size and traveling speed have been shown to depend on the parameter values (coupling strength, coupling range, refractory period, density of inactive nodes etc.) [31,33,34,35,36,37].

The chimera and bump states recapitulated in the previous paragraph were all obtained using identical elements on all nodes of the network and identical non-local linking with equal and time-independent weights. To keep the connectivity geometry identical in all nodes and to avoid boundary value effects all simulations were performed with periodic boundary conditions in one, two or three dimensions [3,38]. In the present study, we impose dynamical updating on the links themselves and investigate its influence on the formation of chimera and bump states in LIF networks. As an exemplary case, we use bistable couplings evolving via diffusive non-local interactions, to be described in detail in the next sections.

In earlier studies, multistability was introduced already in the structural parameters characterizing the intrinsic dynamics of oscillators/nodes. In multistable systems, the dynamics drive the system to one of multiple possible asymptotic steady states. One special case of multistability is bistability, where the possible asymptotic steady states are only two. Former attempts in this direction include the introduction of bistability in the internal frequency [39] or in the amplitude [40] of the oscillators. As a result, complex frequency and amplitude chimera states were produced as well as frequency-amplitude entanglement effects [40]. In the present study we introduce bistability in the bonding between the different nodes in the network. One example of synaptic multistability in neuroscience relates to the neuron-glial interactions in the brain. In neuron-glial networks synapse—glia (astrocyte) signaling could modify the synaptic weight and can induce low or high firing regimes [41,42]. Its biological meaning is that the interactions with the glial cells may modify the strength of the connections between neurons and these connections may take specific values, leading initially random coupling values to multiple fixed points. Other examples are the hippocamplal excitatory synapses which exhibit bistability as part of their role in learning and memory. Long-term potentiation and long-term depression mechanisms in these (excitatory) synapses can lead to stable high or low strengths [43]. Neuromodulation substances (hormones) can also transform the intrinsic firing properties of circuit neurons by altering the effective synaptic strength and often massively altering the network synaptic properties [44]. In all above examples, various factors drive different domains in the network to acquire different coupling parameters and these influence the local firing rates and the internal dynamics. We will demonstrate in the following sections that the effects of introducing a 3rd-order interaction mechanism may lead to bistability (simplest case of multistability) in the coupling strengths and further, for specific parameter domains, to coexistence of bump-like and chimera-like regions in the same network. This co-habitation of bumps and chimeras simultaneously on the network comes as a direct result of the bistable time-dependent coupling mechanism and, to the best of our knowledge, has not been previously observed. Other interesting effects include (a) the shift of the coupling parameters, where non-trivial composite chimera-like or bump-like dynamics are observed, (b) memory effects, in the sense that the final spatial distribution of coupling strengths recalls traces of their initial distribution and (c) the presence of a transition point in the coupling range, where bistability terminates abruptly giving rise to single fixed point oscillatory dynamics.

We should stress here that the bistable plasticity rules proposed here are not to be confused with Hebbian like adaptivity, where plasticity depends on the pre- and post-synaptic potentials following the Hebb’s principle that “neurons that fire together, wire together” or other spike-timing-dependent plasticity (STDP) models [45,46,47,48,49,50]. Rather, in the present study plasticity is inherent in the structure of the neuron axons which are considered to be bistable (or more generally, multistable) and they get adjusted according to the general tendencies of link weights in the neighboring neural environment [42,51].

All studies proposed above find potential applications in the complex synchronization patterns observed in the brain. In particular, hybrid synchronization patterns have been recorded at the onset of epileptic seizures, where the transition from the asynchronous state to full synchronization is mediated by phenomena of local synchronization [52,53,54,55]. Another situation where hybrid synchronization patterns are relevant is the unihemispheric sleep of birds and mammals. Migratory birds, during their long-distance flights, they sleep with one eye open, meaning that one part of their brain is in the dormant state while the other part is awake. This situation can be viewed as a hybrid state of the brain. Similarly, mammals (dolphins, seals) are taking rest with one eye open to look out for danger. In addition, dolphins need to remain conscious even when they are sleeping because their breathing is not automatic. Therefore, part of their brain remains at the conscious state to control breathing even when the other part is at the dormant state. This way, the dolphin brain also operates at a hybrid state [55,56,57,58]. All points discussed in the preceding paragraphs regarding the existence of excitatory and inhibitory links in the brain as well as the plasticity in the couplings are also relevant in the cases of these real-world situations.

In the next section, we present the single/uncoupled LIF model, the ring LIF network and the plasticity rules employed. In Section 3, we study the formation of complex chimera-like states under bistability in the link plasticity and in Section 4, we examine the influence of link plasticity on the formation of bump-like states. In both Section 3 and Section 4, we provide evidence and stress the presence of spatial memory effects. In Section 5, we investigate the effects that link plasticity induces on the network dynamics; namely, we show that bistability on the link weights causes cohabitation of chimera-like states and bump-like states on the ring network. In Section 6, entropy values are used for the quantification of the network dynamics. Using the local entropy values as quantitative index, we show that for large coupling ranges *R*, the system transits from bistable to homogeneous, while the transition *R*-values depend on the coupling parameters. In the Conclusions section, we recapitulate our main results and discuss open problems. To facilitate the reading of this manuscript and to highlight the influence of plasticity in the network, in the two Appendices A.1 and A.2 sections we recapitulate some results on the presence of chimera or bump states in LIF networks without plasticity rules.

## 2. The Model

In this section, we first present the dynamics of a single uncoupled LIF neuron (Section 2.1) while in Section 2.2 we present the coupled LIF dynamics implemented on a 1D ring geometry as will be used in the simulations. The difference of this approach from previous studies in the literature lies in the choice of the coupling terms, which here become time-dependent, adjusting to the neighboring coupling environment. At the end, Section 2.3, we briefly discuss entropic measures used to quantify the degree of synchronization of the different observed patterns.

### 2.1. The Uncoupled LIF Dynamics

Historically, the first Integrate-and-Fire neuron models were introduced by Louis Lapicque at the turn of the 20th century to describe the response of nerve fibers to electrical stimuli [59,60,61]. The variable u(t) introduced by Lapicque describes the neuron membrane potential which grows linearly due to the interaction with other neurons, up to a threshold potential, $u_{\mathrm{th}}$. After reaching $u_{\mathrm{th}}$ the *u*-variable (membrane potential) is automatically reset to its rest state value, $u_{\mathrm{rest}}$, forming a periodically firing oscillator. A leaky term (proportional to the potential u(t)) was introduced to inherently avoid potential divergences in the long time scales and to match experimental observations. The version which includes the leaky term is called “Leaky Integrate-and-Fire” neuron model or simply LIF. The many variations of integrate-and-fire models are very popular amongst computational neuroscientists due to their simple dynamics allowing to consider many thousands up to millions of coupled neurons to mimic realistic natural systems. The choice of the variant depends on the particular application. In the present study the LIF model is employed and the dynamics of the single LIF neural oscillator reads as:

(1a) $$\frac{du(t)}{dt}=\mu -u\left(t\right)$$

(1b) $$\lim_{\epsilon \to 0}u\left(t+\epsilon \right)\to u_{\mathrm{rest}},\;\;\;\mathrm{when}\;\;u\left(t\right)\geq u_{\mathrm{th}}\;\;with\;\;u_{\mathrm{th}}<\mu .$$

Equation (1a) represents the integration phase of neuronal activity when the potential increases. At the end of this phase, when the potential reaches its threshold value $u_{\mathrm{th}}$, the neuron fires and its potential resets to $u_{\mathrm{rest}}$ as described by the event-driven algebraic condition Equation (1b). As the firing is instantaneous, the integration phase restarts immediately after the resetting. Biological neurons have an additional refractory phase after firing. During this refractory period the neurons do not integrate their potential, independently of whether or not they are uncoupled or they are connected in a network and receive input from other neurons or external sources. This is because biological neurons typically undershoot after firing an action potential, i.e., their potential takes a value below the resting potential and then slowly “recovers”. Because of the undershooting they are less likely to fire during this period. To keep the system as simple as possible, in this study we will not consider a refractory period but leave this extended model for future studies.

Within the interval $\left[u_{\mathrm{rest}},u_{\mathrm{th}}\right]$ the solution to Equations (1a) and (1b) can be obtained analytically and the period $T_{s}$ that the single LIF oscillator takes to fire after rest is calculated as [27]

(2) $$\begin{array}{c} T_{s}=\ln\left[\frac{\mu -u_{\mathrm{rest}}}{\mu -u_{\mathrm{th}}}\right]. \end{array}$$

### 2.2. The Coupled LIF Dynamics with Bistable Plasticity

When many LIF elements are coupled on a ring network, the differential equations which describe the evolution of the potential $u_{i}\left(t\right)$ of a neuron at position *i* on the ring of *N* neurons are provided below in Equations (3a) and (3b). Because the coupling strengths are also time dependent, an additional equation, Equation (3c), is necessary to describe the evolution of the coupling strengths, $\sigma_{i}\left(t\right),\;\;i=1,N$.

(3a) $$\frac{du_{i}\left(t\right)}{dt}=\mu -u_{i}\left(t\right)+\frac{1}{2R}\sum_{j=i-R}^{i+R}\sigma_{i}\left(t\right)\left[u_{j}\left(t\right)-u_{i}\left(t\right)\right]$$

(3b) $$\lim_{\epsilon \to 0}u_{i}\left(t+\epsilon \right)\to u_{\mathrm{rest}},\;\;\;\mathrm{when}\;\;u_{i}\left(t\right)\geq u_{\mathrm{th}}\;\;with\;\;u_{\mathrm{th}}<\mu$$

(3c) $$\frac{d\sigma_{i}\left(t\right)}{dt}=c_{\sigma }\left(\sigma_{i}-\sigma_{l}\right)\left(\sigma_{i}-\sigma_{c}\right)\left(\sigma_{i}-\sigma_{h}\right)+\frac{s}{2R}\sum_{j=i-R}^{i+R}\left[\sigma_{j}\left(t\right)-\sigma_{i}\left(t\right)\right].$$

In Equations (3a), (3b) and (3c), the parameter $u_{\mathrm{th}}$ is identical for all neurons: when reaching it they reset to their rest potential $u_{\mathrm{rest}}$ following identical event-driven algebraic conditions, Equation (3b). μ is the value that the potential of any neuron would asymptotically tend to, if there was no resetting condition; therefore, to achieve periodic firing $u_{\mathrm{th}}$ must satisfy the condition $u_{\mathrm{th}}<\mu$. Each neuron interacts with all other neurons within a distance *R*, which is also known as the coupling range. I.e., in the ring geometry every neural oscillator *i* is linked with all other oscillators *j* in the range i−R≤j≤i+R with time-dependent coupling strength $\sigma_{i}\left(t\right)$. All node indices in Equations (3a), (3b) and (3c) are taken modN, where *N* is the number of nodes in the ring network.

The non-local coupling term at the right hand-side of Equation (3a) represent only electrical synapses or gap junctions. Such synapses have been previously considered in Integrate-and-Fire networks, often together with chemical synapses [62,63,64,65]. To keep our model as minimal as possible, we only address gap junction type of synapses in this study which are known to enhance synchrony and we keep the case of chemical and combined chemical and electrical synapses for future studies. An additional limitation of the LIF model is that the action potentials are not directly modeled, but are implicitly assumed to take place during the resettings. Taking into account explicitly the action potentials in the simulations is important, because the coupling terms are expected to grow large during action potentials. This additional feature is also left for future studies.

The coupling strengths in Equation (3c) are assumed to evolve in time via a nonlinear equation of order three, since the highest nonlinearity comes from the term $\left(\sigma_{i}-\sigma_{l}\right)\left(\sigma_{i}-\sigma_{c}\right)\left(\sigma_{i}-\sigma_{h}\right)$ and is of order $\sigma_{i}^{3}$. All other terms’ contributions are of order $\sigma_{i}^{2}$ or lower. The value of the constant $c_{\sigma }$ in Equation (3c) governs the evolution of the coupling strengths. The constants $\sigma_{l}$ (standing for $\sigma_{\mathrm{low}}$), $\sigma_{c}$ (standing for $\sigma_{\mathrm{center}}$), and $\sigma_{h}$ (standing for $\sigma_{\mathrm{high}}$), denote the three fixed points of Equation (3c) in the absence of the coupling term and, without loss of generality, we assume that $\sigma_{l}<\sigma_{c}<\sigma_{h}$. In the same equation, *s* is a parameter that controls the strength of the interaction term in the time-dependent couplings. In this version of link plasticity each neuron/node *i* has its own time-dependent coupling strength $\sigma_{i}\left(t\right)$ with the neighbors, while the parameters μ, $u_{\mathrm{th}}$, $u_{\mathrm{rest}}$, *R*, $\sigma_{l}$, $\sigma_{c}$, $\sigma_{h}$ and *s* are common to all network elements.

As stated in the Introduction, the plasticity rule introduced in Equation (3c) is related to the evolution of the various link weights under the influence of their neighboring connectivity. Namely, the evolution of the connectivity $\sigma_{i}$ is influenced by the link weights of the neighbors at positions *j*, where i−R≤j≤i+R. This coupling plasticity is not to be confused with Hebbian adaptivity rules, where the evolution of link weights are considered with respect to the potential dynamical variables, $u_{i}$ and $u_{j}$ [45,46,47,48].

As in the case of single neurons, coupled neurons also undergo a refractory period after firing. The refractory period, denoted by $T_{r}$, is of the order of $T_{s}$, i.e., it accounts for approximately half the period of the single neurons but also depends on the type of neural cells. Earlier studies have shown that hybrid (chimera or bump) states emerge even in the absence of a refractory period [25,27,37,66]. Since in coupled systems the refractory period is not essential for the development of chimera or bump states, it will not be considered hereafter.

**Figure 1:**
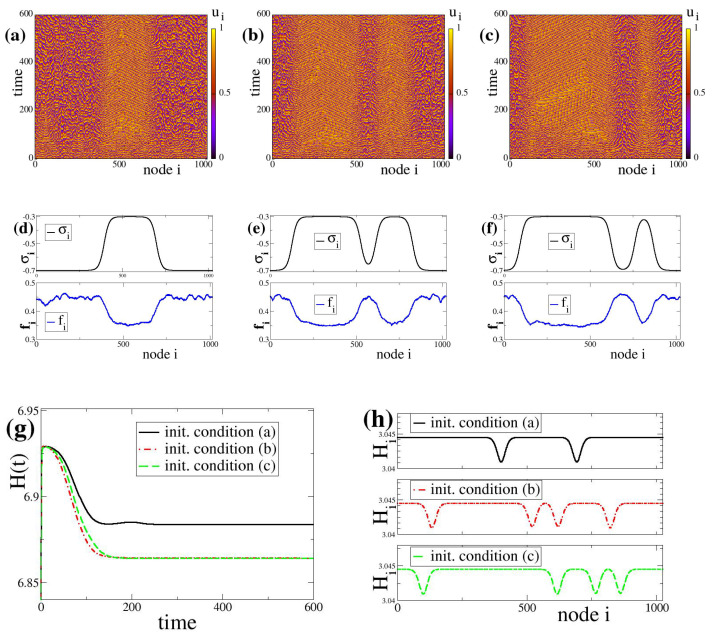
Coupled LIF dynamics with bistable plasticity and negative coupling strengths. Top row: spacetime plots of the potential values $u_{i}$ starting from three different initial conditions are depicted in panels (**a**–**c**). Middle row: (**d**–**f**): Corresponding asymptotic coupling strengths $\sigma_{i}$ and firing rates $f_{i}$ for the above three initial conditions. Bottom row: (**g**) The global entropy evolution with time, H(t) and (**h**) The local entropy values $H_{i}$ at the final stages of the simulations. In panels (**g**,**h**) the black solid lines correspond to initial condition (**a**), the red dashed-dotted line to (**b**) and the green dashed line to (**c**). The simulations in (**a**–**c**) start from different random initial conditions in $u_{i}$ and $\sigma_{i}$. All other parameter values are identical in the three cases: μ=1, $u_{\mathrm{rest}}=0$, $u_{\mathrm{th}}=0.98$, N=1024, R=10, $\sigma_{l}=-0.7$, $\sigma_{c}=-0.5$, $\sigma_{h}=-0.3$, $c_{\sigma }=-1.0$ and s=0.9.

**Figure 2:**
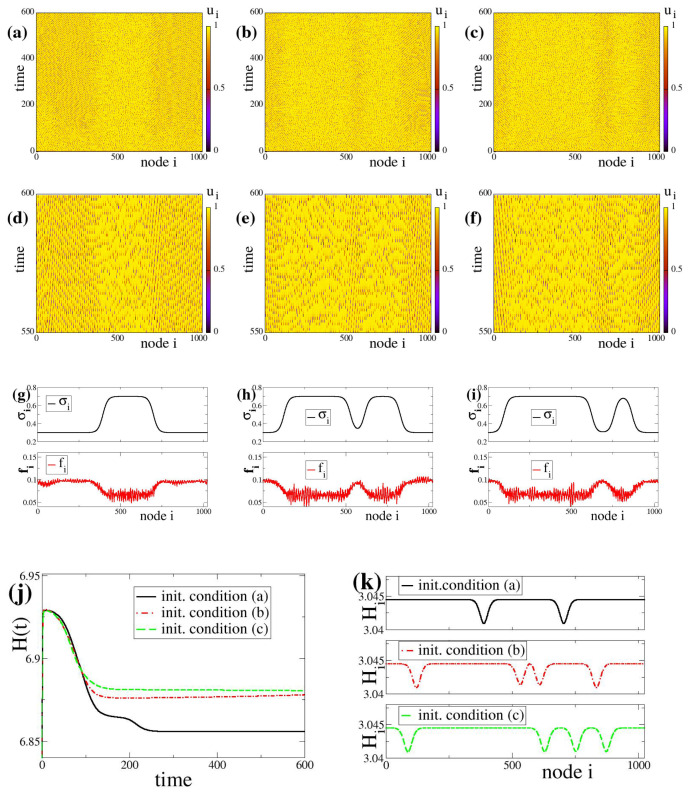
Coupled LIF dynamics with bistable plasticity and positive coupling strengths. Typical potential spacetime profiles $u_{i}$ for three different initial conditions (**a**–**c**) forming bump-like states. For better understanding of the bump-like state complexity, in the second row, panels (**d**–**f**) show specific details in restricted time scales between 550–600 TUs of panels (**a**–**c**), respectively. Third row: (**g**–**i**): Corresponding coupling strengths $\sigma_{i}$ and firing rates $f_{i}$ for the above three initial conditions. Forth row: (**j**) The global entropy evolution in time, H(t) and (**k**) The local entropy values $H_{i}$ at the final stages of of the simulations. In panels (**j**,**k**) the black solid lines correspond to initial condition (**a**), the red dashed-dotted line to (**b**) and the green dashed line to (**c**). The simulations in (**a**–**c**) start from different random initial conditions in $u_{i}$ and $\sigma_{i}$. Coupling fixed point values are: $\sigma_{l}=+0.3$, $\sigma_{c}=+0.5$ and $\sigma_{h}=+0.7$. All other parameter values are the same as in Figure 1.

In the simulations, aiming to keep the system as generic as possible, we consider uniformly and randomly distributed initial conditions both for the potentials, $u_{i}\left(t=0\right)$ and coupling strengths, $\sigma_{i}\left(t=0\right)$. Namely, the initial states are randomly sampled from uniform distributions such that $0\leq u_{i}\left(t=0\right)<u_{\mathrm{th}}$ and $-1\leq \sigma_{i}\left(t=0\right)\leq 1$. The system size needs to be chosen large enough, usually N≥1000 nodes, to approach asymptotic dynamics avoiding effects related to finite system sizes. To treat all elements equally and avoid boundary effects we use periodic boundary conditions as also discussed in the Introduction. The linear chain containing the *N* oscillators closes forming a ring. Because of the periodic boundary conditions all indices are considered modN, i.e., for the potential $u_{i+N}\left(t\right)=u_{i}\left(t\right)$ and similarly for the coupling strengths $\sigma_{i+N}\left(t\right)=\sigma_{i}\left(t\right)$, for i≤N. Typical spacetime plots of the network evolution can be viewed in Figure 1a–c for negative fixed points $\left(\sigma_{l},\sigma_{c},\sigma_{h}\right)$ and Figure 2a–c for positive fixed points. Detailed description of these patterns and their properties will follow in Section 3 for Figure 1 and in Section 4 for Figure 2.

### 2.3. Quantitative Measures

To quantify the complexity of synchronization patterns induced by the nonlinearity in dynamics together with the plasticity of the bondings the most frequently used measure is the average firing rate, or average frequency, $f_{i}$, which counts the number of firings of neuron *i* per unit time [3,38]. Namely, if the oscillator *i* has performed $Q_{i}$ resettings in the time interval ΔT, the average firing rate is calculated as:

(4) $$f_{i}=\frac{Q_{i}}{\Delta T}=\frac{1}{T_{i}},\;\;\;i=1,\cdots N.$$

In Equation (4), $T_{i}$ is the average period of oscillator *i*, which may differ substantially from $T_{s}$ due to the interactions in the network. Similarly for the average firing rates, $f_{i}$. For the correct estimation of the firing rates, ΔT needs to be sufficiently large compared to the firing periods of isolated LIF neurons. The average firing rates account for the network evolution and dynamics and they can differentiate between homogeneous and hybrid states only in the case of static(immobile) chimeras or bumps. In the case of chimera states, if the coherent and incoherent domains travel in the network, all network elements spend some amount of time in the coherent motion while for other time intervals they perform incoherent motion. This way, for long time averages all elements acquire a common average firing rate. The same is true for mobile bumps. This is a main disadvantage in the use of the firing rates for distinguishing a traveling chimera state from a purely homogeneous oscillatory network, and similarly for traveling bump states.

In the domain of static and dynamical pattern formation the image entropy has been previously employed to identify chimera states in complex coupled maps [67]. Here, the global entropy, H(t), is used to identify the evolution and stability of bumps or chimera states. H(t) is here defined in terms of the homogeneity/heterogeneity of the coupling strengths as:

(5a) $$H\left(t\right)=-\sum_{i=1}^{N}{\tilde{\sigma }}_{i}\left(t\right)\log{\tilde{\sigma }}_{i}\left(t\right)$$

(5b) $$\tilde{\sigma_{i}}\left(t\right)=\frac{|\sigma_{i}\left(t\right)|}{\sum_{j=1}^{N}\left|\sigma_{j}\left(t\right)\right|}.$$

Because the values of $\sigma_{i}$ can take arbitrary values, greater or less than unity and with positive or negative signs, for the calculation of the local and global entropies all coupling values need to be normalized. (Note that the local and global entropies are regarded as measures of diversity and organization in the system). In addition, in case of negative $\sigma_{i}$ (inhibitory linking) the absolute values of $\sigma_{i}$ are used in Equations (5a) and (5b) to calculate H(t) (and later on in Equation (6) for the calculations of the local entropies). The global entropy, H(t), can not distinguish between moving and immobile chimera and bump states, for the same reasoning as explained above in the case of the average firing rates.

The local entropy evolution, $H_{j}\left(t\right)$, around node *j* offers information on how the entropy changes locally in time and is calculated as:

(6) $$H_{j}\left(t\right)=-\sum_{k=j-R}^{j+R}{\tilde{\sigma }}_{k}\left(t\right)\log{\tilde{\sigma }}_{k}\left(t\right).$$

As in the case of the global entropy, in Equation (6) for the calculations of the local entropy the normalized weights are used and the absolute values of $\sigma_{i}$ are taken if $\sigma_{i}$ are negative (inhibitory linking).

Other quantitative measures include the size distribution P(σ) of the link weights, the fraction of elements (relative size of) that belong to the active and subthreshold domains [27,37], the distributions of firing rates and link weights. As will be discussed in the next sections, the quantitative measures depend on the model parameters, on the connectivity scheme and on the coupling strengths.

In the next sections, the following working parameter set is used which is known to give rise to chimera and bump states in the absence of plasticity: μ=1, $u_{\mathrm{rest}}=0$, $u_{\mathrm{th}}=0.98$, N=1024. The parameters which are varied for the exploration of the network regimes are $\sigma_{l}$, $\sigma_{c}$, $\sigma_{h}$ and *R*. For the network integration the forward Euler scheme is used with integration step is $dt=10^{-3}$ Time Units (TU). Runge-Kutta integration is also employed in some cases for confirmation of the results. The integration mostly takes place for 5000 TUs. For the calculation of the firing rates, $f_{i},\;i=1,\cdots N$, the first 1000 TU are considered as transient and are ignored.

## 3. The Formation of Composite Chimera-Like States Due to Bistable Linking Dynamics

As first examples, we plot in Figure 1a–c the spacetime plot of the system, Equations (3a), (3b) and (3c), starting with three different initial conditions both in the $u_{i}$ and $\sigma_{i}$ variables. All other system and network parameters are the same for all nodes. After the transient states, the three initial conditions drive the system to distinct final states which contain domains with different firing rates and σ-distributions. In particular, in Figure 1a the system develops one large domain of low firing rates coexisting with a smaller domain of high firing rates. In Figure 1b,c, the systems develop two domains of low firing rates coexisting with two domains of high firing rates, the size of the different domains being variable in Figure 1b,c. The above simulations indicate that the introduction of plasticity in the links introduces a kind of memory effects because the final hybrid states develop domains of different sizes, reflecting the particularities of the initial distribution of the $\sigma_{i}$ values. That is why, starting from different initial conditions, under the same (identical) parameter values, we end-up in spatially different σ-distributions (different number of domains, different sizes of domains), as can be observed in Figure 1 (top row). That is to say, different basins of attraction with respect to initial conditions may end-up in different fixed points of the (multistable or bistable) dynamics and the finally formed domains keep a record of the (basin of attraction of) the initial conditions where they originated from. Therefore, the use of the term “memory effect” here is not related to the memory used in neuroscience, brain science or AI. It is used as in dynamical systems, where the system at the asymptotic state retains (keeps the “memory” of ) some properties (patterns, features, correlations) of its initial state.

We need to stress here that the fixed points $\left(\sigma_{l},\sigma_{c},\sigma_{h}\right)=\left(-0.7,-0.5,-0.3\right)$ used in the present simulations take all negative values, giving rise to oscillatory dynamics. Because the different σ-values influence locally the system dynamics, we observe chimera-like states. Note that in the absence of link-weight evolution (no plasticity, $c_{\sigma }=s=0$) and for constant and identical linking between all elements, the final chimera states of the LIF model develop coherent and incoherent domains whose sizes do not depend on the initial conditions, $u_{i}\left(t=0\right)$, and only the position of the domains may be displaced in the network [27,28]. For a quick comparison of the LIF network evolution with and without coupling plasticity, we add in the Appendix A.1 typical spacetime plots of the system evolution under different coupling parameters as indicated on the plots.

Regarding the firing rates, for the three different initial conditions shown in Figure 1a–c the firing rates $f_{i}$ and the coupling strength $\sigma_{i}$ distributions are shown in Figure 1d–f, respectively. The spatial distributions of $f_{i}$ and $\sigma_{i}$ also reflect the formation of domains in the system, corroborating the observations in the spacetime plots related to the memory effects, as discussed in Section 2.2.

These memory effects are further reflected in the evolution of the global entropy which also depends strongly on the initial conditions. As an example, we plot in Figure 1g the entropy evolution in the three cases of identical parameters while starting from different initial conditions. We observe that each initial condition (a, b or c) leads to a final state with different entropy. Note that in the case of constant and equal $\sigma_{i}$ values, then ${\tilde{\sigma }}_{i}=1/N$. Consequently, a constant value of the entropy is expected only in the case of equipartition and in this case *H* only depends on the system size as H=logN. For the present system, N=1024 and therefore H=6.93 for constant and equal $\sigma_{i}$, independently of the system parameters. We note that during transient all initial conditions reach *H*-values close to 6.93 but at the final state they all drop considerably below 6.9, indicating certain degrees of organization.

The local entropy $H_{i}$ at the final state of the system for the three initial conditions is plotted in panel h of Figure 1. We may observe that the local entropy drops at the points that correspond to transitions between the different domains. Using Equation (6) for the case of constant values of $\sigma_{i}$ in different local domains of the system, when the domain size is 2R+1 the local entropy has the value $H_{i}=\log\left(2R+1\right)$. For R=10 used in Figure 1, the local entropy values become $H_{i}=3.0445$. Indeed, such values appear in regions of constant $\sigma_{i}$ values, see the corresponding areas in Figure 1a–c as well as in Figure 1d–f.

In regions of transition between domains of different σ-values the local entropy drops, as demonstrated by the variations in the $H_{i}$ in Figure 1h. These drops delineate the borders between the different domains of coherence, while the number of entropy maxima or minima can be used to quantitatively count the number of coherent/incoherent domains in the chimera-like state. The local entropy can be also viewed as an indicator of plasticity and is also linked to the memory of the initial distribution of σ-values.

As a general remark on the formation of chimera-like states in the LIF network, we would like to point out that the final states of the network are highly influenced by the form of Equation (3c) governing the evolution of the link-weights. Due to the explicit bistability in Equation (3c) it is expected that two types of weights ($\sigma_{l}$ and $\sigma_{h}$) are favored in the system. However, it is not guaranteed that (spatial) domains of low and high link sizes will be formed. This comes as a cooperative result due to interactions between nodes. Later on, in Section 6, the *R*-parameter region where these cooperative effects manifest will be discussed.

The results in the present section and in Appendix A.1 are all obtained under negative (inhibitory) coupling strengths. As will be discussed in the next section, Section 4, and in Appendix A.2, in the cases of positive coupling strengths (eg., $\left(\sigma_{l},\sigma_{c},\sigma_{h}\right)=\left(0.3,0.5,0.7\right)$ fixed points) bump and bump-like states will be observed.

## 4. The Formation of Composite Bump-Like States Due to Bistable Linking Dynamics

In this section, we report on the effects of bistable plasticity in the case of positive fixed points $\sigma_{h}$, $\sigma_{c}$ and $\sigma_{l}$ in the dynamics of the coupling strengths. In parallel to the case of negative (inhibitory) couplings, here we perform numerical integration of Equations (3a), (3b) and (3c) for positive (excitatory) coupling strengths with $\sigma_{l}=+0.3$, $\sigma_{c}=+0.5$ and $\sigma_{h}=+0.7$, starting with three different random initial conditions in $u_{i}$ and $\sigma_{i}$. All other parameters are identical to the ones in Figure 1 following the working parameter set. The results are shown in Figure 2.

Typical spacetime plots of the potentials $u_{i}$ for three different random initial conditions in $u_{i}$ and $\sigma_{i}$ are presented on the top row of Figure 2. All other parameters are identical in the three plots. The time interval is here extended from t=0 to 600 TUs and covers the transient time. In all three cases, Figure 2a–c, the network nodes spend considerable time near the threshold value (yellow regions) while only occasionally they oscillate (as shooting solitaries) and reset to the rest state. The occasional activity of shooting solitary nodes is not uniform throughout the system. One may discern in Figure 2a a middle region where the density of solitaries is lower, while to the left and the right the density is higher. In Figure 2b,c (different initial conditions), two areas of low solitary activity are formed, whereas in Figure 2c the size of the two areas of low solitary density are significantly different. This heterogeneous activity in a network composed by identical and identically linked elements is also a result of spontaneous breaking of the spatial symmetry and is characteristic of bump states, see also Ref. [27] and Appendix A.2. Because the bump states here (Figure 2) are formed under time-dependent plasticity conditions, where asymptotically the system has reached a state of variable coupling weights, these states will be called “bump-like” states. These bump-like states are composed of domains where different solitary densities cause different average firing rates on a silent background of subthreshold elements (yellow regions in Figure 2a–f).

For better inspection of the appearance and disappearance of the occasional activity in the system, in Figure 2d–f we present details of the Figure 2a–c, respectively. In the 2nd row, the time interval is restricted to the last 50 time units, *t* = 550–600 TU. The different activity regimes are hard to discern in Figure 2a–f but they are better visible in the firing rate and coupling strength diagrams. Figure 2g–i presents the coupling strength and the firing rate spatial distributions starting with the different random initial conditions. We note that in the case of Figure 2 where coupling strengths are positive, the firing rates are considerably lower than in Figure 1 where the coupling strengths are negative. These lower firing rates are attributed to the tendency of the nodes to stay at subthreshold values for long time intervals and to occasionally perform resettings to the rest state. This has also previously been reported in the case of time-independent (constant) couplings and for positive coupling strengths, see Ref. [37] and Appendix A.2.

Memory effects are also evident here. Comparing Figure 2a–c and corresponding quantitative indices Figure 2g–i, we understand that the initial (random) spatial distributions of $\sigma_{i}$ have given rise to steady states with different domain sizes, influenced by the specific initial $\sigma_{i}\left(t=0\right)$ states. We recall that in case of identical linking sizes ($\sigma_{i}=\sigma =const.$ and without plasticity) and different initial conditions $u_{i}\left(t=0\right)$, when chimera states or bump states are formed the distribution of domain sizes remain statistically constant, independent of the randomly chosen initial conditions, $u_{i}\left(t=0\right)$.(The term “statistically constant” implies that if we start from different initial conditions the system will converge to an asymptotic state exhibiting the same quantitative characteristics within the bounds of error (statistics).)

Regarding the entropy evolution in the network, in Figure 2j, we present the evolution of the global entropy in time for the three different initial conditions. We observe similar approaches to the steady state as in the case of inhibitory dynamics, but here, the entropy levels attained are different and the first initial condition shows the lowest final entropy (compare with Figure 1g).

With respect to local entropy, changes in the $H_{i}$ values occur at the transition regions between active bumps and subthreshold domains, see Figure 2k. Similarly to the case of inhibitory coupling, the drops in the local entropy permit to count (quantitative index) the number of active bumps and subthreshold domains of the bump pattern. As in Section 3, the local entropy profile also retains some characteristics of the original spatial distribution of the links (memory effects). In addition, time-dependent plasticity has caused the shift of bump-like state formation toward shorter coupling ranges *R* (in Figure 2 R=10). This shift in the formation of complex synchronization patterns along with the shifts shown in the case of chimera-like states are further discussed in Section 6.

Similarly to the case of chimera-like states, in the cases of positive couplings the final states of the network are also highly influenced by the form of Equation (3c) governing the evolution of the link-weights, leading to the domination of two types of weights. And although the position of the high or low σ could be random, the cooperativity (interactions leading to concerted behavior) between nodes results also in the formation of spatial domains of low and high link sizes. The *R*-parameter region where these spatial domains are formed is further discussed in Section 6.

## 5. Coexistence of Chimera-Like and Bump-Like States Due to Bistable Linking Dynamics

**Figure 3:**
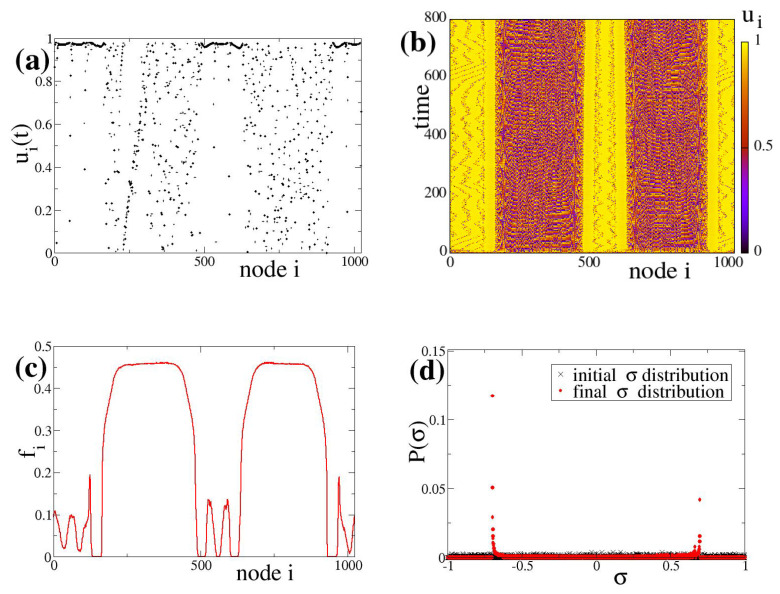
LIF network with bistable plasticity coupling comprising positive and negative fixed points cause the formation of complex synchronization patterns combining both bump and chimera features. (**a**) Typical snapshot of the neuron potential $u_{i}\left(t\right)$ (*t* = 800 TU), (**b**) spacetime plot, (**c**) the average firing rate $f_{i}$ and (**d**) the distribution of coupling strengths σ at t=0 TU (black crosses, homogeneous random initial conditions) and at t=800 TU (red dots). Parameter values are: μ=1, $u_{\mathrm{rest}}=0$, $u_{\mathrm{th}}=0.98$, N=1024, R=40, $\sigma_{l}=-0.7$, $\sigma_{c}=0.0$, $\sigma_{h}=+0.7$, $c_{\sigma }=-1.0$ and s=0.9. Simulations start from random uniform initial conditions in $u_{i}$ and $\sigma_{i}$.

Starting with random coupling strengths distributed on the various nodes of the network (in addition to random initial potentials $u_{i}\left(t=0\right)$), we now concentrate on an exemplary case where the fixed points in the dynamics of the coupling weights (Equation (3c)) take mixed positive and negative values. Mixed excitatory and inhibitory links are well known to coexist in the same network, notably in the brain dynamical networks [10]. We use the working parameter set (see Section 2.3), with appropriate values of $\sigma_{l}$, $\sigma_{c}$, $\sigma_{h}$ and *s*. The plots in Figure 3 are representative complex hybrid patterns in the presence of synaptic plasticity with mixed positive and negative fixed points.

More specifically, in Figure 3, we present typical results of the complex hybrid patterns produced when synaptic plasticity is considered in the coupling strength with $\sigma_{l}=-0.7$, $\sigma_{c}=0.0$, and $\sigma_{h}=+0.7$. Previous studies have revealed that for negative (inhibitory) coupling strengths chimera states are supported by the LIF network [27,28], while bump states appear for positive (excitatory) couplings [37]. By considering evolutionary dynamics leading to bifurcation with positive and negative fixed points in the coupling, at the asymptotic state of the LIF network we record hybrid behavior as indicated in Figure 3a. In this panel we note coexistence of two incoherent domains with domains where subthreshold oscillations persist. Within the subthreshold domains we can record small active bumps. This is further clarified in the spacetime plot of Figure 3b; there, we may first note the presence of yellow subthreshold regions supporting small bumps which move stochastically to the left and right on the ring. The subthreshold regions are separated by active incoherent domains characterized by different $u_{i}$ values. The positions of the active incoherent and the subthreshold domains on the ring stay fixed in time. In fact, the erratically traveling small bumps in the yellow (subthreshold) regions do not cross the incoherent domains, but they are scattered back by them. In Figure 3c, we record the firing rates and we note that the active domains demonstrate the highest firing frequencies, while the bump-like regions show lower frequencies. The difference in the firing frequencies between bumps and the incoherent domains may be attributed both to the occasional firing and to the motion of the former ones. As the bumps move erratically to the left and right, the energy is transmitted from one element to the neighbors and the firing changes position on the network in time. This means that the elements spend part of their time at the subthreshold state and part in the active state. Because the firing rates are recorded as averages on the nodes over many time units, non-zero firing values are recorded over all nodes affected by the motion of bumps, while the frequency of firings is lower than if the bumps were localized. As a result, we record lower firing activity inside the subthreshold regions on the positions of the bumps than in the incoherent regions that are always active. In Figure 3d, we plot with black crosses the initial distribution P(σ)(t=0) of the coupling strengths $\sigma_{i}$ and with red dots the long time (asymptotic) steady state distribution, P(σ) (t=800 TU). Because the coupling strengths were initially chosen randomly and homogeneously in the interval (−1.0, 1.0), the initial distribution of $\sigma_{i}$ is flat. At the steady state of the network, the distribution P(σ) becomes bimodal, with maxima at $\sigma ∼\sigma_{l}=-0.7$ and $\sigma ∼\sigma_{h}=0.7$, as formatted by the bistable Equation (3c).

Another observation worth mentioning is the difference in height in the two maxima of the final bimodal distribution P(s). Indeed, the maximum amplitude corresponding to inhibitory coupling, P(σ∼−0.7)=0.12, is higher than the excitatory coupling one, P(σ∼+0.7)=0.04. This is not unexpected, since the active chimera-like regions cover a larger number of nodes than the active bumps in the subthreshold regions. Which of the two domains (chimera-like regions or subthreshold regions) will dominate in the final state is, again, a matter of initial conditions.

To the best of our knowledge, complex synchronization patterns composed by bump-like and chimera-like states co-habitating on the network is a direct effect of bistable plasticity and have not been observed before in simulations of neuron networks. Furthermore, it would be interesting to investigate whether or not phenomena such as the coexistence of stable chimera domains and bump domains in the network can be linked with local synchronization phenomena observed in the brain, in view of the fact that both inhibitory and excitatory links are known to be present in the healthy brain.

## 6. The Influence of the Coupling Range on Synaptic Bistability

As earlier discussed, one of the effects of synaptic bistability is the presence of chimera-like or bump-like states for small values of the coupling range *R*. We also recall that in these parameter regions (R<<N) spatially hybrid states are not formed for constant or homogeneous linking, see also Appendices A.1 and A.2. To explore the *R*-regions where chimera-like or bump-like states are formed due to synaptic bistability, we perform numerical simulations of LIF networks for inhibitory and excitatory coupling and *R* ranging from 1 to 80 lattice units. For quantifying the presence of hybrid states, we use the average deviation $d_{H}$ of the local entropies $H_{i},\;\;i=1,\ldots \;,N$, from the maximum local entropy $H_{\max}$ at the asymptotic state. The quantity $d_{H}$ is here abbreviated as “local entropy deviation” and its squared value is defined as:

(7a) $$d_{H}^{2}=\frac{1}{N}\sum_{j=1}^{N}{\left[H_{\max}-H_{j}\right]}^{2}.$$

(7b) $$H_{\max}=\max\left\{H_{j}\right\},\;\;\;j=1\cdots N.$$

In Equations (7a) and (7b), the values of $H_{j}$ and $H_{\max}$ are recorded at the asymptotic state, after the transient period.

**Figure 4:**
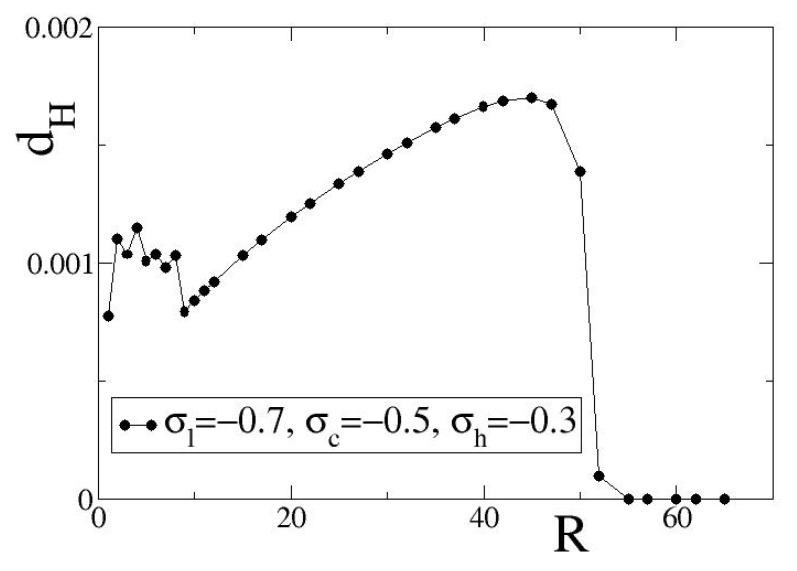
Local entropy deviations $d_{H}$ as a function of the coupling range *R* for inhibitory coupling. Parameter values are: $\sigma_{l}=-0.7$, $\sigma_{c}=-0.5$, $\sigma_{h}=-0.3$. Other parameters are as in Figure 1. All simulations start from the same random uniform initial conditions both in $u_{i}$ and in $\sigma_{i}$.

In Figure 4, we present variations of $d_{H}$ with *R* for inhibitory coupling, using as coupling fixed points the exemplary set $\left(\sigma_{l},\sigma_{c},\sigma_{h}\right)$ = (−0.7,−0.5,−0.3). The fixed points in the selected set take all negative values leading to inhibitory asymptotic dynamics. Other parameters as in the working parameter set. The solid black line with black circles in Figure 4 serves as guide for the eyes.

For small values of the coupling range, R≤10, we observe non-monotonous variations of $d_{H}$ as a function of *R*. We note that for R=1 we have the limit of diffusion where hybrid synchronization regimes are not observable. In this short range limit, 1≤R≤10, the coupling ranges are very short and any small increase in *R* may influence substantially the spatial distribution of domains where $\sigma_{l}$ or $\sigma_{h}$ dominate. That is the reason of observing the non-monotonous variations in $d_{H}$.

For intermediate coupling distances, we observe a monotonous increase of $d_{H}$ with *R*. As *R* increases, organization takes place in larger and larger domains and this is reflected in the index $d_{H}$. For even larger values of *R*, one of the two stable fixed points prevails and the spatial distribution of link weights becomes homogeneous. As a result, all weights become equal and, therefore, $d_{H}\to 0$. The local entropy deviation $d_{H}$ is here used to characterize the degree of spatial organization of the coupling strengths and, hence, it can play the role of an order parameter.

The shape of $d_{H}$ vs. *R* curve in Figure 4 is typical for general inhibitory parameter values, but the *R*-value where the transition from bistability to monostability (one single fixed point) occurs depends on the precise values of the fixed points $\left(\sigma_{l},\sigma_{c},\sigma_{h}\right)$. For the exemplary set (−0.7,−0.5,−0.3), the transition occurs at R∼55, while for (−0.8, −0.5, −0.1) at R∼90. These transition values also vary for different initial conditions, but the transition at the points where $d_{H}\to 0$ is always sharp for single simulations, as in Figure 4. In the Supplementary Material, average $d_{H}$ values over 10 different initial conditions are provided. The difference in the *R* value where the transition occurs can be dependent also on the length of the interval between the attracting fixed points, which for the case of set (−0.7,−0.5,−0.3) is 0.4, while for set (−0.8,−0.5,−0.1) is 0.7.

**Figure 5:**
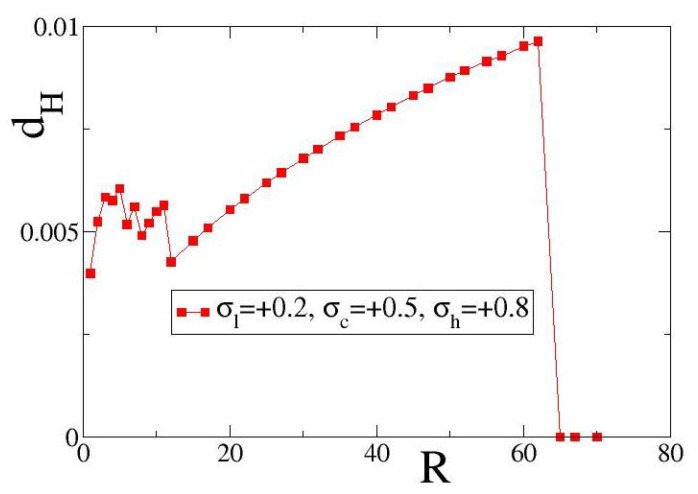
Local entropy deviations $d_{H}$ as a function of the coupling range *R* for excitatory coupling. Parameter values are: $\sigma_{l}=+0.2$, $\sigma_{c}=+0.5$, $\sigma_{h}=+0.8$. Other parameters are as in Figure 1. All simulations start from the same random uniform initial conditions both in $u_{i}$ and in $\sigma_{i}$.

In Figure 5, we examine the case of positive fixed points and present variations of the local entropy deviation $d_{H}$ with the coupling range *R* for excitatory coupling. As exemplary set of coupling fixed points, we use $\left(\sigma_{l},\sigma_{c},\sigma_{h}\right)$ = (+0.2, 0.5, +0.8) (the solid red line is set as guide for the eyes). Other parameters are as in Figure 4.

As in the case of Figure 4a, in Figure 5a we also observe non-monotonous variations of $d_{H}$ vs. *R* in the small coupling ranges, 1≤R≤17. The reason is similar to the case of negative fixed points, namely, for small *R*-values any increase in *R* may influence substantially the spatial distribution of domains where $\sigma_{l}$ or $\sigma_{h}$ dominate. Similarly, for intermediate coupling ranges we observe a monotonic increase of $d_{H}$ vs. *R* up to an *R*-value where transition occurs and one of the two fixed points, $\sigma_{l}$ or $\sigma_{h}$, dominates, leading again to an homogeneous link-weight distribution. For the present parameter values and initial conditions, the critical value of *R* where the transition occurs is 67. In the Supplementary Material, average $d_{H}$ values over 10 different initial conditions are presented. Apart from the difference in the transition coupling range, another difference between Figure 4 and Figure 5 is in the scales of $d_{H}$ values: the $d_{H}$ in Figure 5 are approximately one order of magnitude higher than those in Figure 4.

Comparing Figure 4 and Figure 5, we conclude that the general form of the $d_{H}$ vs. *R* curve is essentially conserved in the case of bistable plasticity with positive or negative couplings. While the form of the curve is conserved for an extended region of parameter values, the precise *R* and $d_{H}$ ranges where the non-monotonous behavior or monotonous increase dominate as well as the *R*-values where $d_{H}$ drops to 0 (transition point) depend strongly on the specific coupling parameter values and, in particular, in the values and the signs of the three fixed points $\left(\sigma_{l},\sigma_{c},\sigma_{h}\right)$.

## 7. Conclusions

In the present study, we concentrate on the influence of bistable plasticity rules in the dynamics of LIF networks. Due to the presence of bistable dynamics in the equation that governs the coupling evolution, it is expected that two (high and low) coupling values will prevail in the system. However, it is not guaranteed that spatial domains of high and low couplings will be formed. The spatial domains emerge here as a result of cooperativity in the network.

Previous studies have demonstrated the presence of chimera states for inhibitory coupling strengths and bump states for excitatory couplings in nonlocally connected LIF networks. In the case of inhibitory coupling, we show that bistable synaptic rules may shift (lower) considerably the region of coupling ranges where chimera-like states are observed. Similarly, for the positive couplings, the introduction of bistability in the synaptic strengths lowers considerably the *R* ranges where bump-like states occur. In addition, we show that for appropriate choice of the stable synaptic fixed points, it is possible to obtain cohabitation of chimera-like and bump-like regions simultaneously on the network.

To quantify the presence of the hybrid states in the network, we use the local entropy deviations $d_{H}$. Using $d_{H}$ as an index of synaptic organization, we numerically show that there is a transition point in the coupling range values *R* where the system transits from bistable to single fixed point dynamics. The transition is abrupt and takes place at intermediate coupling ranges. While before the transition the network presents a distribution of local entropies around two fixed points, at the transition one of the fixed points disappears and, thereafter, all local entropies become equal. The form of $d_{H}$ vs *R* retains its qualitative features across a wide range of parameters; however, the specific *R*-values where the abrupt transition takes place depend strongly on the coupling parameters.

Memory effects have also been recorded, where the spatial organization of the final state of the network contains recollection of the original (t=0) distributions of link weights. Therefore, starting from different initial conditions we may end up in final states with statistically different spatial ordering/organization, eventhough all system parameters are kept to the same values.

Direct extensions of this work may include the study of the *R*-transition as a function of the distance between the stable fixed points $\sigma_{l}$ and $\sigma_{h}$ or the coupling strength *s*, as well as various aspects of cohabitation of chimera-like and bump-like states simultaneously on the network.

Future extensions may include the possibility of multistable linking $\sigma_{ij}\left(t\right)$ which depends both on the pre- and post-synaptic neurons. Other, Hebbian-like plasticity rules [45,46] or spike-timing-dependent plasticity [68] may contain nonlinear linking terms depending on the values of the involved potentials $u_{i}$ and $u_{j}$ on the last term of the right-hand side in Equation (3c). In a different direction, yet, we may consider the influence of a power law distribution on the number of links emanating from the neurons of the network or/and power-law distribution of the link weights. This conforms with recent biomedical studies reporting that the number of synapses per axon follow long-tailed distributions [69] and is in line with Hebb’s propositions of long-range distributions in the links per node [45]. This structural property has been recently attributed to the property of preferential attachment of the neurons in the network [70].

As discussed in Section 2.2, in the present study we only consider non-local couplings in the network, which assimilate electrical, gap-junction synapses. To keep the coupling scheme minimal, chemical synapses were not considered in this studies. In future studies we plan to address the combined effects of chemical and electrical synapses focusing on both cooperative and competitive phenomena. 

## Figures and Tables

**Figure A1:**
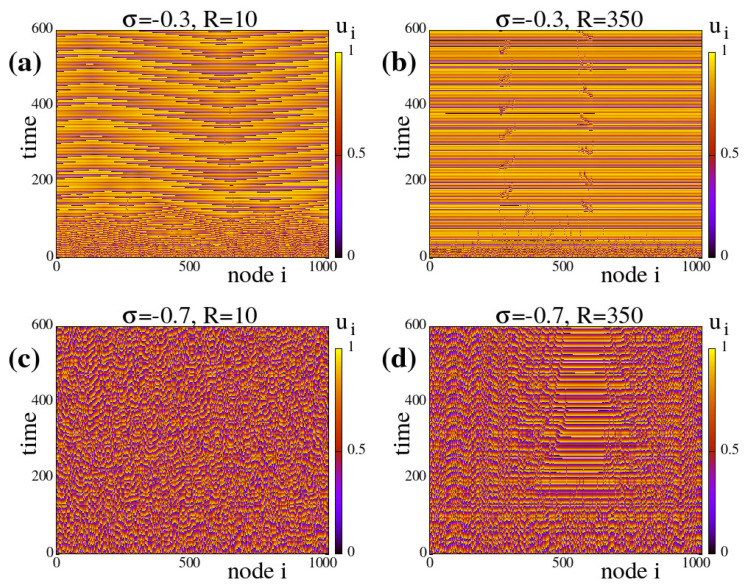
Synchronization patterns of the potential values $u_{i}\left(t\right)$ for inhibitory coupling without link plasticity. Typical spacetime plots for (**a**) σ=−0.3 and R=10, (**b**) σ=−0.3 and R=350, (**c**) σ=−0.7 and R=10, and (**d**) σ=−0.7 and R=350. All other parameter values are as in Figure 1 of the main text. Simulations start from the same random initial conditions in $u_{i}\left(t=0\right)$.

**Figure A2:**
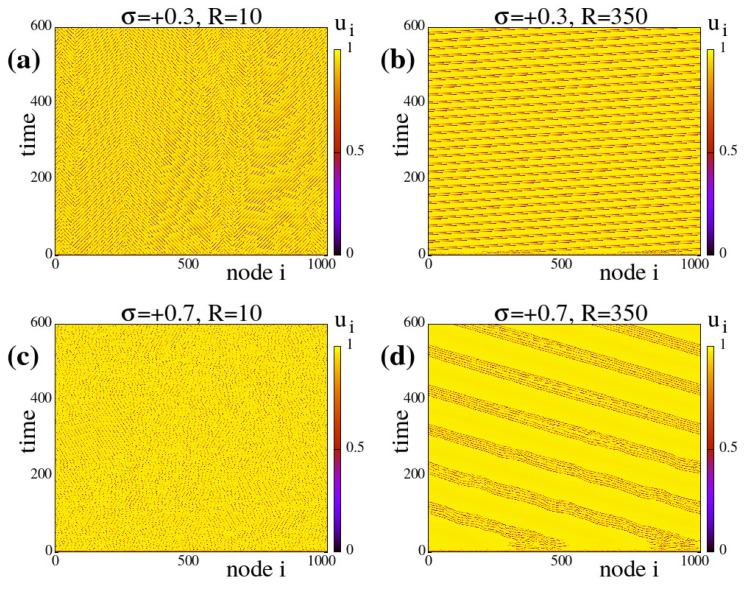
Synchronization patterns of the potential values $u_{i}\left(t\right)$ for excitatory coupling without link plasticity. Typical spacetime plots for (**a**) σ=0.3 and R=10, (**b**) σ=0.3 and R=350, (**c**) σ=0.7 and R=10, and (**d**) σ=0.7 and R=350. All other parameter values are as in Figure 1 and in Appendix A.1, Figure A1. Simulations start from the same random initial conditions in $u_{i}\left(t=0\right)$.

## Data Availability

The data supporting the conclusions of this article will be made available by the authors on request.

## Supplementary Materials

The following supporting information can be downloaded at: https://www.mdpi.com/article/10.3390/e27030257/s1, and contains a brief report of the local entropy deviations averaged over many (10) initial conditions.

## Appendix A

In the two appendices below, we recapitulate some results from earlier publications on the LIF model without plasticity rules. Basically, we integrate Equations (3a) and (3b), while keeping all coupling strengths $\sigma_{i}$ to a constant value σ. The constant σ’s are negative in the case of inhibitory coupling (see Appendix A.1) and positive in the case of excitatory coupling (see Appendix A.2). These results may be found scattered in previous publications, notably in [27,28,37]. Here, we provide a short recapitulation of these results for the convenience of the readers and for direct comparison of the LIF networks with and without plasticity rules.

The equations which now describe the system with time-independent (constant) couplings are:

(A1a) $$\frac{du_{i}\left(t\right)}{dt}=\mu -u_{i}\left(t\right)+\frac{\sigma }{2R}\sum_{j=i-R}^{i+R}\left[u_{j}\left(t\right)-u_{i}\left(t\right)\right]$$

(A1b) $$\lim_{\epsilon \to 0}u_{i}\left(t+\epsilon \right)\to u_{\mathrm{rest}},\;\;\;\mathrm{when}\;\;u_{i}\left(t\right)\geq u_{\mathrm{th}}\;\;with\;\;u_{\mathrm{th}}<\mu .$$

With respect to Equation (3a), Equation (A1a) has coupling strength σ which is constant in time and takes a value common to all nodes. Equation (3c) is redundant (not needed) here, since all coupling strengths do not evolve (remain constant) in time.

### Appendix A.1. Results for LIF Network Without Plasticity Rules: Inhibitory Coupling

In this section, we present the system evolution without coupling plasticity and for negative values of the coupling strength (inhibitory coupling). For comparative reasons, we will present spacetime plots for σ=−0.3 and −0.7 with R=10 and R=350. All other parameters are the same as the ones used in the main text.

In Figure A1a, we present the spacetime plot of the system for σ=−0.3 corresponding to the first of the fixed points in the coupling strength (as in Section 3 and Figure 1) and R=10. We see that for homogeneous and short-distance coupling the system, after transient, reaches the homogeneous steady state. For these couplings strengths (σ=−0.3), only for long-distance couplings non-trivial effects arise such as coexistence of coherent and incoherent domains, see Figure A1b where σ=−0.3 and R=350. [Nevertheless, careful observations of Figure A1a may spot the presence of a single solitary state around i=650, where the node escapes the altogether coherent environment. Solitary states are related to the birth of chimera states].

Similarly, in Figure A1c, we present the spacetime plot of the system for smaller value σ=−0.7 corresponding to the second attractive fixed point in the coupling strength presented in Section 3 and Figure 1. The coupling range is small, R=10, as in Figure A1a and in the other cases of plasticity studied in the main text. We note a certain degree of inhomogeneous firing in the system without indications of coherence. For this coupling strength, typical chimera states develop when the coupling range takes large enough values, see Figure A1d with σ=−0.7 and R=350. For a more complete study on the presence of chimera states for negative coupling strengths and different parameter values in the LIF model without plasticity, we refer the interested reader to Refs. [27,28].

### Appendix A.2. Results for LIF Network Without Plasticity Rules: Excitatory Coupling

In analogy with the presentation in the previous Appendix A.1, we here recall results on the system evolution without coupling plasticity and for positive values of the coupling strength (excitatory coupling).

In this case (σ>0 identical for all nodes), the system presents bump states where active elements appear in a background of silent subthreshold elements. Namely, for small values of the coupling range, we observe erratically appearing and moving active elements which operate as shooting solitaries, see the cases of R=10 in Figure A2a,c for σ=+0.3 and +0.7, respectively. As *R* increases, the active nodes organize in domains which travel around the ring with constant velocity. The size of the active traveling domains and their velocity depend on the parameters *R* and σ, while their direction (left-wise or right-wise) is determined by the initial conditions. Typical examples of traveling bumps are presented in Figure A2b,d. These figures indicate that the size of the active traveling bumps increases with σ. Further discussions on studies on bump states in 1D LIF networks without time-dependent plasticity are presented in Ref. [37].
