# Supplementary material for: Multistable Synaptic Plasticity Induces Memory Effects and Cohabitation of Chimera and Bump States in Leaky Integrate-and-Fire Networks
Source: Entropy (Basel). 2025 Feb 28;27(3):257. doi: 10.3390/e27030257 (PMC11941565; doi:10.3390/e27030257)
Supplement: Supplementary file 1 [file entropy-27-00257-s001.zip › entropy-3306516-supplementary.pdf]

Supplementary Material to Figs. 4 and 5

In the Supplementary Material we present the curves d_H vs R averaged over 10 different initial conditions. In Fig. Suppl1, the results of $<d_H>$ vs R are shown for σ_l =-0.7, σ_c =-0.5 and σ_h =-0.3.

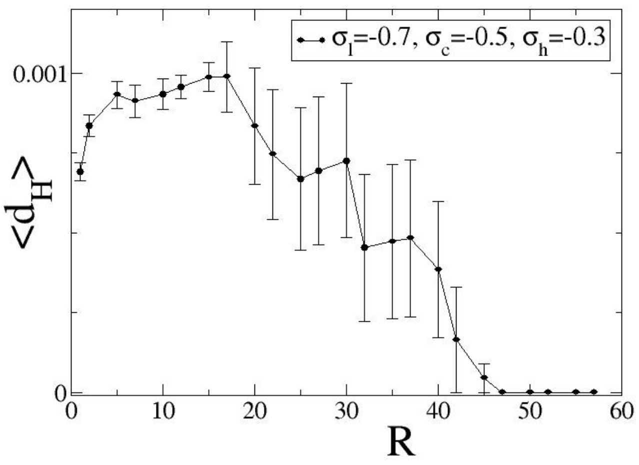

Fig. Suppl1. Supplemental plot to Fig.4 averaged over 10 different initial conditions.

In Fig. Suppl1, we note that for small values of R we have error bars of small sizes, indicating that the d_H take similar values with small variations. As the R values increase in some of the simulations the d_H values remain finite and in others they drop abruptly to 0. That is why the error bars increase significantly. Finally, for large values of R all simulations lead to single fixed point in the σ-values and all d_H as well as the errors vanish.

A similar scenario is also followed for the case of positive coupling strengths, see Fig. Suppl2.

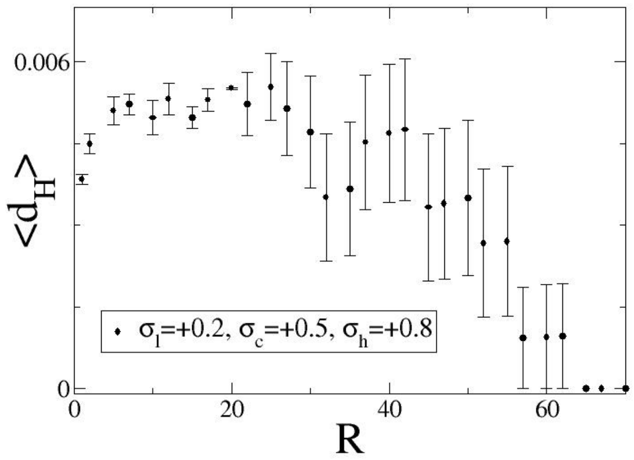

Fig. Suppl2. Supplemental plot to Fig.5 averaged over 10 different initial conditions.

In Fig. Suppl2, the results of $<d_H>$ vs R are shown for σ_l =-0.7, σ_c =-0.5 and σ_h =-0.3 and are averaged over 10 different initial conditions. As in the case of Fig. Suppl1, small error bars are recorded for small values of R as different simulations show similar distributions of σ-values. As the R values increase in some of the simulations the $d_H \rightarrow 0$ while others have finite values. Finally, in the large R-values the σ's take a single values and therefore $<d_H>=0$ in all simulations.

Note that there is a difference of one order of magnitude in the values of $<d_H>$ recorded in Figs. Suppl1 and Fig. Suppl2, as was also the cases in Fig. 4 and Fig. 5 in main text.
